# Detailed analysis of sputum and systemic inflammation in asthma phenotypes: are paucigranulocytic asthmatics really non-inflammatory?

**DOI:** 10.1186/s12890-016-0208-2

**Published:** 2016-04-05

**Authors:** Sophie Demarche, Florence Schleich, Monique Henket, Virginie Paulus, Thierry Van Hees, Renaud Louis

**Affiliations:** Department of Respiratory Medicine, CHU Liege, Sart-Tilman B35, 4000 Liege, Belgium; GIGA I3 Research Group, University of Liege, Liege, Belgium; Department of Clinical and Hospital Pharmacy, University of Liege, Liege, Belgium

**Keywords:** Asthma, Phenotypes, Healthy subjects, Sputum cytology, Blood leukocyte count, CRP, Fibrinogen

## Abstract

**Background:**

The technique of induced sputum has allowed to subdivide asthma patients into inflammatory phenotypes according to their level of granulocyte airway infiltration. There are very few studies which looked at detailed sputum and blood cell counts in a large cohort of asthmatics divided into inflammatory phenotypes. The purpose of this study was to analyze sputum cell counts, blood leukocytes and systemic inflammatory markers in these phenotypes, and investigate how those groups compared with healthy subjects.

**Methods:**

We conducted a retrospective cross-sectional study on 833 asthmatics recruited from the University Asthma Clinic of Liege and compared them with 194 healthy subjects. Asthmatics were classified into inflammatory phenotypes.

**Results:**

The total non-squamous cell count per gram of sputum was greater in mixed granulocytic and neutrophilic phenotypes as compared to eosinophilic, paucigranulocytic asthma and healthy subjects (*p* < 0.005). Sputum eosinophils (in absolute values and percentages) were increased in all asthma phenotypes including paucigranulocytic asthma, compared to healthy subjects (*p* < 0.005). Eosinophilic asthma showed higher absolute sputum neutrophil and lymphocyte counts than healthy subjects (*p* < 0.005), while neutrophilic asthmatics had a particularly low number of sputum macrophages and epithelial cells. All asthma phenotypes showed an increased blood leukocyte count compared to healthy subjects (*p* < 0.005), with paucigranulocytic asthmatics having also increased absolute blood eosinophils compared to healthy subjects (*p* < 0.005). Neutrophilic asthma had raised CRP and fibrinogen while eosinophilic asthma only showed raised fibrinogen compared to healthy subjects (*p* < 0.005).

**Conclusions:**

This study demonstrates that a significant eosinophilic inflammation is present across all categories of asthma, and that paucigranulocytic asthma may be seen as a low grade inflammatory disease.

**Electronic supplementary material:**

The online version of this article (doi:10.1186/s12890-016-0208-2) contains supplementary material, which is available to authorized users.

## Background

The technique of induced sputum has been pivotal in the emergence of the concept of inflammatory asthma phenotypes. Although it is technically demanding and time-consuming, several centers have applied the technique of induced sputum to characterize asthma inflammatory phenotypes in routine [1–7]. It has been suggested that airway inflammation may be subdivided into four phenotypes according to the level of granulocyte airway infiltration: eosinophilic, neutrophilic, mixed granulocytic and paucigranulocytic [2, 3]. This latter has been considered as a non-inflammatory type of asthma as the sputum analysis of these patients was suggested not to differ from healthy subjects [8].

These inflammatory phenotypes have been analyzed with respect to their demographic, functional and clinical characteristics in several studies [2, 3, 9, 10]. However, there has been fewer studies looking in detail at sputum cells [2], blood leukocytes [11], systemic inflammatory markers or at these variables in combination in a large cohort of asthmatics classified according to the extent of airway granulocytic inflammation, and investigating how those groups compared with healthy subjects.

Here, in a retrospective analysis, we report on sputum cell counts, blood leukocytes, C-reactive protein (CRP) and fibrinogen in a large series of asthmatics seen in daily practice of a University Clinic and compare the results with those in healthy subjects.

Our results show that paucigranulocytic asthmatics may display a low grade airway and systemic inflammation.

## Methods

### Study design, setting and participants

We conducted a retrospective cross-sectional study on asthmatic patients and healthy subjects recruited from the University Asthma Clinic of Liege. Asthmatic patients were eligible for the study if they had a first visit with a successful sputum analysis between 1 October 2003 and 5 February 2015. The diagnosis of asthma was based on the presence of typical symptoms (wheezing, breathlessness, chest tightness, cough) and at least one of the following: forced expiratory volume in one second (FEV_1_) increase of ≥12 % and 200 ml after inhalation of 400 μg salbutamol or a provocative concentration of methacholine causing a 20 % fall in FEV_1_ (PC20M) less than 16 mg/ml. Atopy was defined by the presence of at least one positive specific IgE (>0.35 kU/L; Phadia; Groot-Bijgaarden, Belgium) to one or more common aeroallergen (cat, dog, grass pollen, tree pollen, house dust mite and a mixture of moulds). Fractional exhaled nitric oxide (FENO) measurements were performed at 50 ml/s of flow rate (NIOX, Aerocrine, Sweden). Healthy subjects were recruited by advertisement and in the hospital staff, during this 12-year period of study. They had no diagnosis of respiratory disease, a FEV_1_ ≥ 80 % and a Tiffeneau index ≥0.7. Only healthy subjects with a successful sputum analysis were selected. This retrospective study was conducted with the approval from the ethics committee of the University Hospital of Liege (Reference 2015/193). Informed consents were obtained from healthy subjects. As for asthmatic patients, all procedures were performed in the context of clinical practice and the retrospective data collection was conducted with the approval from the above-mentioned ethics committee.

### Sputum induction and analysis

Sputum was induced and processed as previously described [12]. Briefly, sputum was induced by hypertonic (5 %) or isotonic saline with salbutamol when post-bronchodilator FEV_1_ was >65 % or ≤65 % predicted, respectively. Sputum was then processed using the whole expectorate technique. Dithiothreitol (DTT) was used as the mucolytic agent. Sputum sample was considered as adequate for cell count when squamous cell count was <80 % [13]. Differential cell count was performed on cytospins after a Diff-Quick staining.

### Asthma phenotypes

Threshold values used to define the eosinophilic and neutrophilic phenotypes were a sputum eosinophil count ≥3 % and a sputum neutrophil count ≥76 %, respectively [14]. The mixed granulocytic phenotype was defined as both raised sputum eosinophil and neutrophil counts and the paucigranulocytic one as sputum eosinophil and neutrophil counts lower than the thresholds.

### Statistical analysis

Results were expressed as frequencies and percentages for categorical variables and as median (interquartile range) or mean ± standard deviation for continuous variables. Comparisons were performed using a Pearson’s chi-squared test for categorical variables, an ANOVA or a Student’s *t*-test for parametric variables, and a Kruskal-Wallis test for non-parametric variables. The Pearson correlation coefficient was used to measure the association between the percentage of sputum neutrophils and eosinophils, and the number of pack-years in asthmatics. For this parametric test, sputum eosinophils were logtransformed and the 0 values replaced by 0.1. A *p* value <0.05 was considered statistically significant. When multiple tests were performed, the statistically significant level was corrected according to the Bonferroni principle. Statistical analysis was done using STATA version 13.0 (Statistical Software, College Station, TX: StataCorp LP).

## Results

### Subject characteristics

The demographic, functional and treatment characteristics of asthmatics and healthy subjects are given in Table 1. In our cohort of asthmatics with a successful sputum analysis (*n* = 833), inflammatory phenotypes were distributed as follows: 42 % of patients were eosinophilic, 16 % neutrophilic, 4 % mixed granulocytic and 38 % paucigranulocytic. The group of neutrophilic asthma presented a higher proportion of women while eosinophilic asthma had a lower proportion of women. Paucigranulocytic asthmatics were the youngest and had the best FEV_1_ (% predicted) and FEV_1_/forced vital capacity (FVC) ratio. All asthma subgroups had a higher prevalence of atopy as compared to healthy subjects (*p* < 0.005: level of statistical significance after Bonferroni correction).

**Table 1:** Demographic, functional and treatment characteristics of asthmatics classified by phenotypes and healthy subjects

	Eosinophilic asthma	Neutrophilic asthma	Mixed granulocytic asthma	Paucigranulocytic asthma	Healthy subjects	*p* value
N (% of asthmatics)	350 (42)	134 (16)	31 (4)	318 (38)	194	-
Women, N (%)	184 (53)	95 (71)^‡†^	19 (61)	197 (62)	105 (54)	0.002
Age, years	49 (34–60)^*^	52 (41–61)^‡*^	61 (43–71)^‡*^	43 (30–54)	45 (27–56)	0.0001
BMI	25.9 ± 4.9^‡^	25.3 ± 5.4	25.5 ± 4.6	26.1 ± 4.8^‡^	24.3 ± 4.1	0.0004
Atopy, N (%)	213 (63)^‡^	69 (52)^‡^	17 (57)^‡^	165 (53)^‡^	46 (28)	<0.001
Smoking status, N (%)						
Non-smokers	190 (54)	73 (55)	19 (61)	162 (52)	109 (57)	>0.05
Current smokers	64 (18)	26 (20)	5 (16)	76 (24)	35 (18)	
Ex-smokers	96 (27)	33 (25)	7 (23)	75 (24)	47 (25)	
FEV_1_, % predicted	81.2 ± 20.9^‡*^	81.6 ± 22.0^‡*^	81.6 ± 20.6^‡^	89.2 ± 18.2^‡^	108.0 ± 13.3	0.0001
FEV_1_/FVC, %	70.7 ± 11.1^‡*^	71.7 ± 11.5^‡*^	70.1 ± 10.5^‡*^	76.2 ± 9.0^‡^	82.1 ± 6.5	0.0001
FENO, ppb	43 (23–82)^*^	20 (13–33)^†§^	36 (21–75)^*^	16 (12–29)	/	0.0001
ICS therapy, N (%)	227 (65)	87 (65)	18 (58)	195 (61)	/	>0.05
ICS dose^a^	500 (0–1000)	800 (0–2000)	800 (0–1600)	400 (0–1000)	/	>0.05
OCS therapy, N (%)	21 (6)	9 (7)	2 (6)	14 (4)	/	>0.05
LABA, N (%)	209 (60)	80 (60)	20 (65)	181 (57)	/	>0.05
LTRA, N (%)	75 (21)	37 (28)	9 (29)	73 (23)	/	>0.05
Theophylline, N (%)	11 (3)	7 (5)	0 (0)	11 (3)	/	>0.05

^‡^ *p* < 0.005, comparison with healthy subjects

^*^ *p* < 0.005, comparison with paucigranulocytic asthma

^†^ *p* < 0.005, comparison with eosinophilic asthma

^§^ *p* < 0.005, comparison with mixed granulocytic asthma

^a^ICS dose in beclomethasone dipropionate - chlorofluorocarbon equivalents

Abbreviations: *BMI* body mass index; *FENO* fractional exhaled nitric oxide; *FEV*_*1*_ forced expiratory volume in 1 s; *FVC* forced vital capacity; *ICS* inhaled corticosteroid; *LABA* long-acting β2-agonist; *LTRA* leukotriene receptor antagonist; *OCS* oral corticosteroid

### Sputum cell counts and viability

**Fig. 1:**
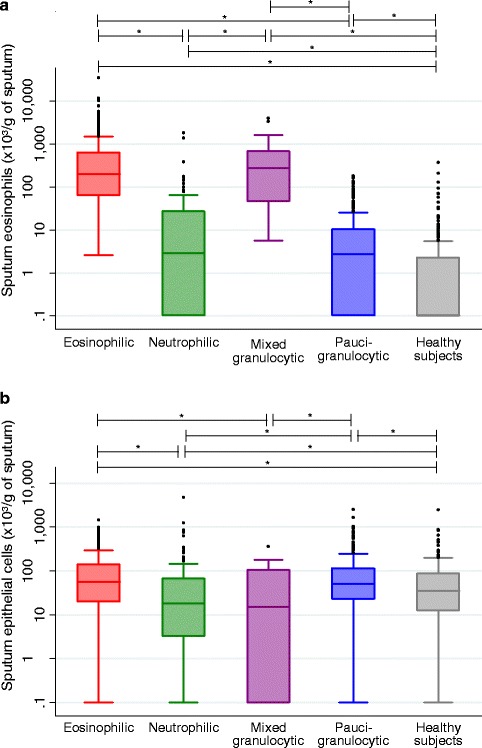
Absolute sputum eosinophils (**a**) and absolute sputum epithelial cells (**b**) in asthma phenotypes and healthy subjects. * *p* < 0.005. Values of 0 were assigned to 0.1 because of the use of a logarithmic scale

The sputum cell counts and viability of asthma phenotypes and healthy subjects are shown in Table 2. The total non-squamous cell count per gram of sputum and the cell viability were greater in mixed granulocytic and neutrophilic phenotypes compared to eosinophilic, paucigranulocytic asthma and healthy subjects. Eosinophilic asthmatics also had significantly greater total sputum cell counts than paucigranulocytic asthmatics and healthy subjects. The fraction of squamous cells was less than 20 % in a majority of the patients, and was particularly low in eosinophilic, neutrophilic and mixed granulocytic asthma. While levels were obviously the highest in eosinophilic and mixed granulocytic phenotypes compared to other asthmatics, sputum eosinophils (in absolute values and percentages) were increased in all asthma phenotypes, compared to healthy subjects (Table 2 and Fig. 1a). Absolute neutrophil counts were increased not only in neutrophilic and mixed granulocytic asthma, but also in eosinophilic asthma subjects, compared with healthy subjects. The group of neutrophilic asthma was the only one to have a lower absolute sputum macrophage count than eosinophilic, paucigranulocytic asthmatics and healthy subjects. Absolute sputum lymphocyte count was greater in eosinophilic asthma (*p* < 0.005, versus paucigranulocytic asthma and healthy subjects), while absolute epithelial cell count was remarkably lower in case of intense neutrophilic inflammation (*p* < 0.005, versus paucigranulocytic and eosinophilic asthma, Table 2 and Fig. 1b).

**Table 2:** Sputum cell counts and viability of asthmatics classified by phenotypes and healthy subjects

	Eosinophilic asthma	Neutrophilic asthma	Mixed granulocytic asthma	Paucigranulocytic asthma	Healthy subjects	*p* value (Kruskal-Wallis test)
	Sp. eosinophils ≥3 %	Sp. eosinophils <3 %	Sp. eosinophils ≥3 %	Sp. eosinophils <3 %		
	Sp. neutrophils <76 %	Sp. neutrophils ≥76 %	Sp. neutrophils ≥76 %	Sp. neutrophils <76 %		
Total non-squamous cell count (× 10^6^/g)	1.18 (0.56–2.85)^‡*^	1.84 (0.82–5.52)^‡*†^	4.10 (1.07–12.00)^‡*†^	0.81 (0.40–2.07)	0.64 (0.37–1.30)	0.0001
Viability (%)	64 (50–75)	79 (68–88)^‡*†^	81 (67–86)^‡*†^	64 (48–75)	67 (53–78)	0.0001
Squamous cells (%)	13 (5–25)^‡*^	12 (3–28)^‡*^	6 (2–23)^‡*^	18 (8–35)	18 (10–31)	0.0001
Eosinophils						
x 10^3^/g	201 (64–636)^‡*^	3 (0–27)^‡†§^	271 (46–685)^‡*^	3 (0–10)^‡^	0 (0–2)	0.0001
% of non-squamous cells	17.1 (7.4–38.0)^‡*^	0.2 (0.0–1.0)^‡†§^	5.2 (3.4–8.8)^‡*†^	0.4 (0.0–1.0)^‡^	0.0 (0.0–0.4)	-
Neutrophils						
x 10^3^/g	335 (135–823)^‡^	1504 (707–4728)^‡*†^	3272 (881–10992)^‡*†^	270 (90–795)	210 (63–563)	0.0001
% of non-squamous cells	33 (16–50)^*^	87 (82–93)^‡*†^	83 (79–88)^‡*†^	42 (21–58)	36 (13–63)	-
Macrophages						
x 10^3^/g	288 (144–648)	169 (52–425)^‡*†^	218 (77–607)	337 (167–853)	285 (120–568)	0.0001
% of non-squamous cells	27 (16–43)^‡*^	9 (4–13)^‡*†^	6 (3–9)^‡*†^	46 (29–61)	46 (30–67)	0.0001
Lymphocytes						
x 10^3^/g	19 (5–52)^‡*^	12 (2–41)	19 (0–88)	10 (3–31)	10 (4–26)	0.0012
% of non-squamous cells	1.7 (0.6–3.2)	1.0 (0.3–1.6)^‡*†^	0.6 (0.0–1.8)^‡†^	1.4 (0.5–2.8)	1.8 (0.8–3.4)	0.0001
Epithelial cells						
x 10^3^/g	56 (20–140)^‡^	18 (3–67)^‡*†^	15 (0–106)^*†^	50 (23–115)^‡^	35 (12–86)	0.0001
% of non-squamous cells	5.0 (2.0–8.6)^*^	1.0 (0.4–2.0)^‡*†^	0.6 (0.2–1.8)^‡*†^	6.0 (2.8–13.0)	4.8 (2.2–11.8)	0.0001

^‡^ *p* < 0.005, comparison with healthy subjects

^*^ *p* < 0.005, comparison with paucigranulocytic asthma

^†^ *p* < 0.005, comparison with eosinophilic asthma

^§^ *p* < 0.005, comparison with mixed granulocytic asthma

Abbreviation: *Sp*. sputum

### Blood leukocyte counts and systemic inflammatory markers

**Fig. 2:**
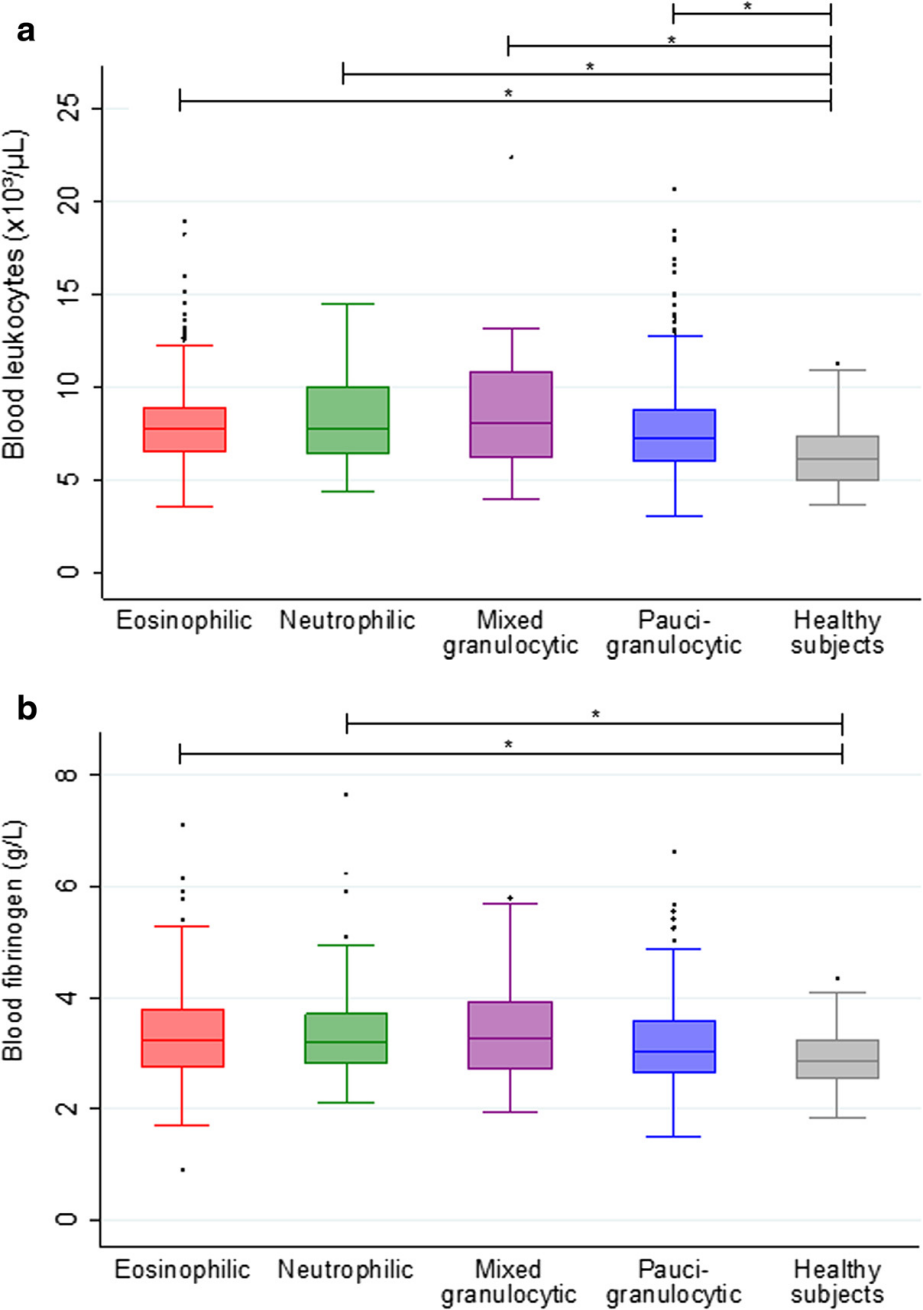
Blood leukocytes (**a**) and blood fibrinogen (**b**) in asthma phenotypes and healthy subjects. * *p* < 0.005

The blood leukocyte counts and systemic inflammatory markers of asthma phenotypes and healthy subjects are shown in Table 3. All subgroups of asthmatics showed an increased level of blood leukocytes compared with healthy subjects (Table 3 and Fig. 2a). As expected, eosinophilic and mixed granulocytic asthmatics had higher blood eosinophils (in absolute values and percentages) than neutrophilic, paucigranulocytic asthma and healthy subjects, but paucigranulocytic asthmatics also showed higher levels of absolute blood eosinophils than healthy subjects. Neutrophilic asthmatics had the highest level of blood neutrophils, compared to eosinophilic, paucigranulocytic asthma and healthy subjects, but both eosinophilic and paucigranulocytic asthmatics also had greater absolute blood neutrophils than healthy subjects. Absolute lymphocyte counts were mainly increased in eosinophilic and paucigranulocytic asthma. Only the group of eosinophilic asthma had increased absolute blood basophil counts compared to healthy subjects. Neutrophilic asthma was characterized by raised CRP and fibrinogen levels, while eosinophilic asthma had raised fibrinogen only, compared with healthy subjects (Table 3 and Fig. 2b).

**Table 3:** Blood leukocyte counts and systemic inflammatory markers of asthmatics classified by phenotypes and healthy subjects

	Eosinophilic asthma	Neutrophilic asthma	Mixed granulocytic asthma	Paucigranulocytic asthma	Healthy subjects^a^	*p* value (Kruskal-Wallis test)
	Sp. eosinophils ≥3 %	Sp. eosinophils <3 %	Sp. eosinophils ≥3 %	Sp. eosinophils <3 %		
	Sp. neutrophils <76 %	Sp. neutrophils ≥76 %	Sp. neutrophils ≥76 %	Sp. neutrophils <76 %		
Blood leukocytes (x 10^3^/μL)	7.76 (6.52–8.87)^‡^	7.75 (6.43–10.00)^‡^	8.02 (6.16–10.83)^‡^	7.25 (6.02–8.75)^‡^	6.12 (5.03–7.37)	0.0001
Blood eosinophils						
/μL	315 (202–513)^‡*^	129 (79–228)^†§^	289 (216–449)^‡*^	140 (85–227)^‡^	106 (68–172)	0.0001
%	4.2 (2.8–6.2)^‡*^	1.7 (1.0–3.0)^†§^	3.9 (2.7–5.8)^‡*^	2.0 (1.1–3.0)	1.8 (1.0–3.0)	0.0001
Blood neutrophils						
/μL	4045 (3204–5140)^‡^	4847 (3260–6323)^‡*†^	4207 (2834–6610)	3971 (3156–5280)^‡^	3416 (2610–4188)	0.0001
%	54.0 (47.6–60.1)^*^	59.5 (52.5–69.3)^*†^	57.6 (44.4–62.4)	56.5 (50.3–62.2)	54.4 (49.6–62.1)	0.0001
Blood monocytes						
/μL	515 (396–667)^‡*^	492 (408–656)^‡^	564 (448–775)^‡^	461 (362–608)	399 (330–550)	0.0001
%	6.7 (5.1–8.3)	6.5 (5.4–8.2)	7.2 (5.6–9.0)	6.4 (5.1–8.2)	7.1 (5.7–8.2)	>0.05
Blood lymphocytes						
/μL	2358 (1918–2809)^‡^	2249 (1765–2807)	2180 (1887–2869)	2337 (1894–2904)^‡^	2009 (1611–2386)	0.0001
%	31.8 (26.0–38.3)	29.4 (22.5–37.1)^‡*^	29.1 (21.0–38.2)	32.7 (27.3–38.2)	33.9 (26.5–37.7)	0.006
Blood basophils						
/μL	42 (29–63)^‡*^	35 (23–51)^†^	39 (27–55)	34 (24–52)	34 (24–46)	0.0001
%	0.6 (0.4–0.8)^*^	0.5 (0.3–0.6)^‡†^	0.5 (0.4–0.7)	0.5 (0.3–0.7)	0.5 (0.4–0.8)	0.0001
CRP (mg/L)^b^	1.4 (0.6–3.6)	1.9 (0.8–5.3)^‡^	2.9 (0.6–8.9)	1.6 (0.6–4.5)	1.1 (0.4–2.4)	0.034
Fibrinogen (g/L)^c^	3.2 (2.8–3.8)^‡^	3.2 (2.8–3.7)^‡^	3.3 (2.7–3.9)	3.1 (2.7–3.6)	2.9 (2.6–3.2)	0.0008

^a^Data available for 96 healthy subjects

^b^Data available for 208 eosinophilic, 92 neutrophilic, 23 mixed granulocytic, 186 paucigranulocytic and 63 healthy subjects

^c^Data available for 194 eosinophilic, 85 neutrophilic, 21 mixed granulocytic, 174 paucigranulocytic and 59 healthy subjects

^‡^ *p* < 0.005, comparison with healthy subjects

^*^ *p* < 0.005, comparison with paucigranulocytic asthma

^†^ *p* < 0.005, comparison with eosinophilic asthma

^§^ *p* < 0.005, comparison with mixed granulocytic asthma

Abbreviations: *CRP* C-reactive protein; *Sp*. sputum

### Analysis of asthma phenotypes according to treatment with inhaled corticosteroids (ICS) and comparison with healthy subjects

For each phenotype, we compared sputum and blood cell counts between patients treated and not treated with ICS and we also compared these subgroups with healthy subjects (Table 4 and Additional file 1: Table S1). In steroid-naïve paucigranulocytic patients, as in the whole group of paucigranulocytic asthma, there was an increase in sputum eosinophil counts, compared to healthy subjects. With respect to systemic inflammation, blood leukocyte, eosinophil and lymphocyte counts were increased in steroid-naïve paucigranulocytic asthmatics, compared to healthy subjects (*p* < 0.0042: level of statistical significance after Bonferroni correction, Table 4). Paucigranulocytic asthmatics receiving inhaled corticosteroids showed an increase in sputum eosinophils and epithelial cells, and an increase in blood leukocyte, neutrophil and lymphocyte numbers (*p* < 0.0042, Table 4). We did not find any statistically significant difference between patients treated and not treated with ICS in the subgroups of paucigranulocytic, neutrophilic and mixed granulocytic asthma (*p* > 0.0042, Table 4 and Additional file 1: Table S1). In eosinophilic asthmatics, patients treated with ICS presented higher levels of sputum epithelial cells, blood leukocytes and blood neutrophils, as compared with eosinophilic patients not treated with ICS (*p* < 0.0042, Additional file 1: Table S1).

**Table 4:** Sputum and blood cell counts of non-eosinophilic phenotypes classified by ICS treatment and healthy subjects

	Paucigranulocytic asthma, not treated with ICS	Paucigranulocytic asthma, treated with ICS	ICS- vs ICS+	Neutrophilic asthma, not treated with ICS	Neutrophilic asthma, treated with ICS	ICS- vs ICS+	Healthy subjects
N	123	195	-	47	87	-	194
Sputum total non-squamous cell count (x 10^6^/g)	0.93 (0.43–2.12)	0.69 (0.36–1.61)	NS	1.45 (0.82–4.65)^‡^	2.10 (0.75–8.02)^‡^	NS	0.64 (0.37–1.30)
Sputum viability (%)	65 (48–78)	64 (48–75)	NS	78 (68–87)^‡^	80 (68–88)^‡^	NS	67 (53–78)
Sputum squamous cells (%)	16 (7–30)	19 (10–37)	NS	18 (5–30)	7 (2–27)^‡^	NS	18 (10–31)
Sputum eosinophils (x 10^3^/g)	4 (0–15)^‡^	2 (0–10)^‡^	NS	2 (0–28)^‡^	4 (0–27)^‡^	NS	0 (0–2)
Sputum neutrophils (x 10^3^/g)	315 (97–994)	245 (86–710)	NS	1197 (707–3776)^‡^	1879 (593–7060)^‡^	NS	210 (63–563)
Sputum macrophages (x 10^3^/g)	451 (188–864)^‡^	280 (154–853)	NS	127 (33–396)^‡^	182 (52–436)	NS	285 (120–568)
Sputum lymphocytes (x 10^3^/g)	12 (3–45)	10 (2–24)	NS	15 (4–41)	11 (1–39)	NS	10 (4–26)
Sputum epithelial cells (x 10^3^/g)	48 (19–113)	52 (25–120)^‡^	NS	19 (4–44)	18 (3–73)	NS	35 (12–86)
Blood leukocytes (x10^3^/μL)^a^	6.87 (5.85–8.51)^‡^	7.47 (6.32–9.06)^‡^	NS	7.87 (6.41–9.93)^‡^	7.71 (6.44–10.06)^‡^	NS	6.12 (5.03–7.37)
Blood eosinophils (/μL)^a^	145 (101–217)^‡^	139 (74–229)	NS	172 (63–293)	123 (80–215)	NS	106 (68–172)
Blood neutrophils (/μL)^a^	3598 (2924–5119)	4211 (3392–5392)^‡^	NS	4804 (3158–6021)^‡^	4885 (3403–6969)^‡^	NS	3416 (2610–4188)
Blood monocytes (/μL)^a^	461 (353–592)	463 (370–615)^‡^	NS	476 (387–648)	519 (419–669)^‡^	NS	399 (330–550)
Blood lymphocytes (/μL)^a^	2352 (1891–2967)^‡^	2321 (1900–2855)^‡^	NS	2518 (1989–3035)^‡^	2167 (1675–2726)	NS	2009 (1611–2386)
Blood basophils (/μL)^a^	35 (25–52)	33 (24–51)	NS	40 (29–53)	33 (22–50)	NS	34 (24–46)

^a^ Data available for 96 healthy subjects

^‡^ *p* < 0.0042, comparison with healthy subjects

Abbreviation: *ICS* inhaled corticosteroid

### Analysis of asthma phenotypes classified with a threshold of sputum eosinophils of 1.01 %

We also used 1.01 % as the threshold of sputum eosinophils to define asthma phenotypes. When using this cutoff value, there was no statistically significant difference in sputum and blood eosinophils (in absolute values and percentages) between neutrophilic or paucigranulocytic asthma, and healthy subjects (the level of statistical significance after Bonferroni correction being a *p* value <0.005, Table 5). However, there was a strong trend for greater absolute sputum eosinophil count in paucigranulocytic asthmatics versus healthy subjects (*p* = 0.005). In the paucigranulocytic asthma subgroup defined with this cutoff value, absolute sputum epithelial cells (50 (24-115) × 10^3^/g), blood leukocytes (7.21 (5.82–8.66) × 10^3^/μL), absolute blood neutrophils (3900 (3083–5299)/μL) and absolute blood lymphocytes (2334 (1891–2904)/μL) were still greater than in healthy subjects (*p* < 0.005).

**Table 5:** Eosinophilic inflammation of asthmatics classified by phenotypes (with a sputum eosinophil cutoff of 1.01 %) and healthy subjects

	Eosinophilic asthma	Neutrophilic asthma	Mixed granulocytic asthma	Paucigranulocytic asthma	Healthy subjects	p value (Kruskal- Wallis test)
	Sp. eosinophils ≥1.01 %	Sp. eosinophils <1.01 %	Sp. eosinophils ≥1.01 %	Sp. eosinophils <1.01 %		
	Sp. neutrophils <76 %	Sp. neutrophils ≥76 %	Sp. neutrophils ≥76 %	Sp. neutrophils <76 %		
N (% of asthmatics)	429 (51)	106 (13)	59 (7)	239 (29)	194	-
Sputum eosinophils						
x 10^3^/g	127 (38–471)^‡*^	0 (0–9)^†§^	77 (31–380)^‡*^	0 (0–5)^‡‡^	0 (0–2)	0.0001
% of non-squamous cells	11.8 (4.0–30.8)^‡*^	0.0 (0.0–0.5)^†§^	3.0 (1.8–6.0)^‡*†^	0.2 (0.0–0.5)	0.0 (0.0–0.4)	0.0001
Blood eosinophils^a^						
/μL	289 (181–444)^‡*^	123 (62–220)^†§^	266 (126–430)^‡*^	133 (78–200)	106 (68–172)	0.0001
%	3.8 (2.4–5.6)^‡*^	1.7 (0.9–3.0)^†§^	2.9 (1.8–5.6)^‡*^	1.9 (1.0–2.7)	1.8 (1.0–3.0)	0.0001

^a^Data available for 96 healthy subjects

^‡^ *p* < 0.005, comparison with healthy subjects

^‡‡^ *p* = 0.005, comparison with healthy subjects

^*^ *p* < 0.005, comparison with paucigranulocytic asthma

^†^ *p* < 0.005, comparison with eosinophilic asthma

^§^ *p* < 0.005, comparison with mixed granulocytic asthma

Abbreviation: *Sp.* sputum

### Analysis according to atopy

The blood and sputum eosinophil counts of asthmatics and healthy subjects according to their atopic status are presented in Table 6. We did not observe any statistically significant difference in these cells between atopic and non-atopic healthy subjects or asthmatics (the level of statistical significance after Bonferroni correction being a *p* value <0.0083).

**Table 6:** Eosinophilic inflammation of asthmatics and healthy subjects according to atopy

	Non-atopic asthmatics	Atopic asthmatics	Non-atopic healthy subjects	Atopic healthy subjects	p value (Kruskal-Wallis test)
N	344	464	118	46	-
Sputum eosinophils					
x 10^3^/g	19 (1–173)^‡*^	34 (3–181)^‡*^	0 (0–3)	0 (0–4)	0.0001
% of non-squamous cells	1.6 (0.2–9.6)^‡*^	2.8 (0.2–13.8)^‡*^	0.0 (0.0–0.4)	0.0 (0.0–0.5)	0.0001
Blood eosinophils^a^					
/μL	188 (109–327)^‡^	217 (124–375)^‡*^	93 (62–135)	134 (80–202)	0.0001
%	2.6 (1.4–4.0)^‡^	3.0 (1.7–4.7)^‡^	1.5 (0.9–2.9)	2.1 (1.6–3.0)	0.0001

^a^Data available for 54 non-atopic healthy subjects and 31 atopic healthy subjects

^‡^ *p* < 0.0083, comparison with non-atopic healthy subjects

^*^ *p* < 0.0083, comparison with atopic healthy subjects

### Analysis according to smoking habits

**Fig. 3:**
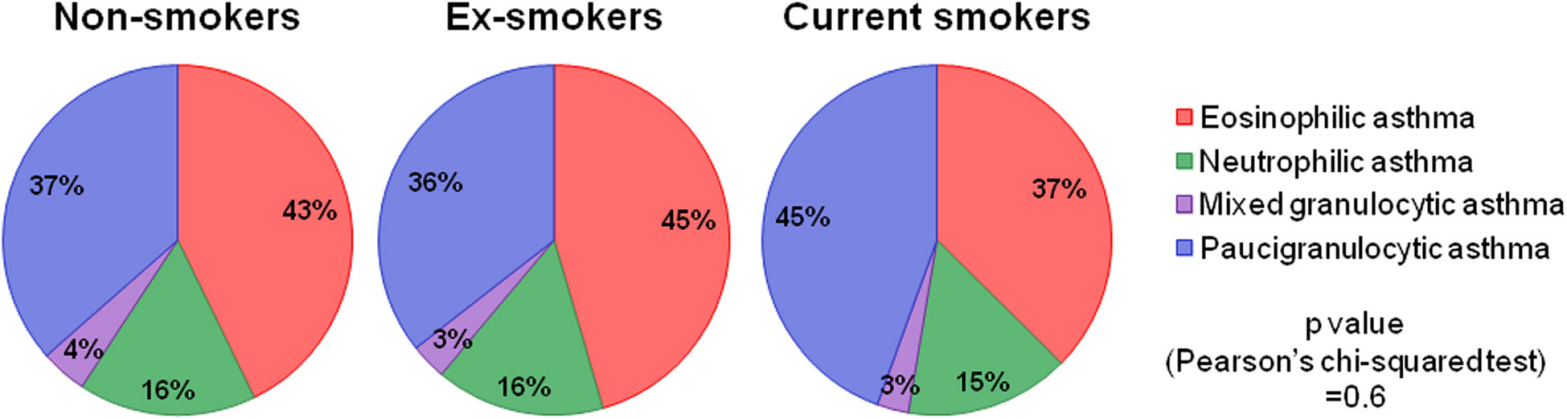
Proportion of asthma inflammatory phenotypes according to the smoking status of asthmatics

**Fig. 4:**
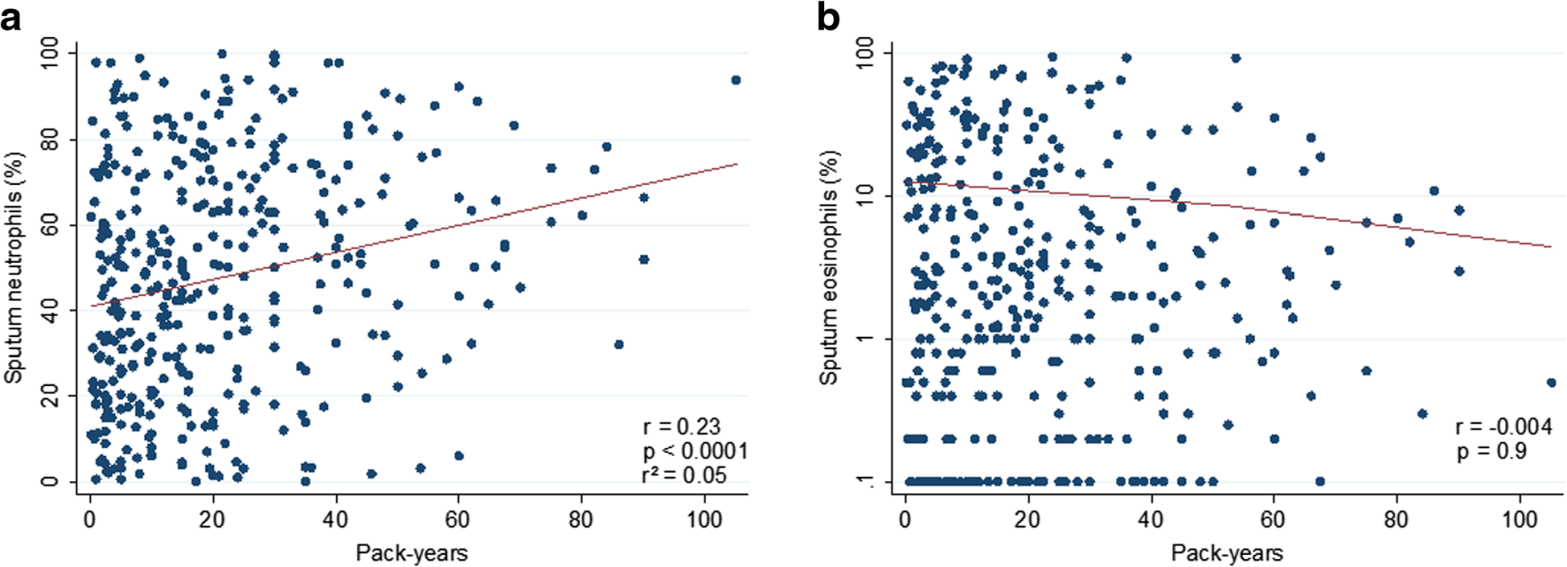
Relationship between sputum neutrophils (**a**) and eosinophils (**b**) and the number of pack-years in ex- and current smoker asthmatics. For sputum eosinophils, values of 0 were assigned to 0.1 because of the use of a logarithmic scale

The proportion of asthma inflammatory phenotypes was analyzed according to the smoking status of patients (Fig. 3), and was not different between non-smokers, ex-smokers and current smokers (*p* = 0.6). There was however a correlation between the number of pack-years and the percentage of sputum neutrophils but this correlation was absent with sputum eosinophils (Fig. 4).

## Discussion

Our study shows that the four inflammatory asthma phenotypes display raised sputum eosinophilia compared to healthy subjects. Eosinophilic asthma showed higher levels of absolute sputum neutrophils and lymphocytes than healthy subjects, while neutrophilic asthmatics had a reduced number of sputum macrophages and epithelial cells. In addition, all phenotypes had increased systemic inflammation reflected by raised total circulating leukocyte counts, mainly accounted for by raised granulocytic blood cell counts compared to healthy subjects.

As previously shown [2], our data indicate that neutrophilic and mixed granulocytic inflammation is more intense than pure eosinophilic and paucigranulocytic ones as the total number of cells per gram of sputum was clearly increased in these two first forms of groups. This might be related to the larger pool of circulating neutrophils and the variety of stimuli able to recruit neutrophils into the airways [15]. Our results showing greater viability in neutrophilic and mixed granulocytic asthma are also in keeping with those reported by Simpson et al. [2]. It may seem paradoxical as neutrophils are recognized to be more fragile than eosinophils in cell culture [16], but this may suggest greater cell turnover and short time of residency of neutrophils in the airways [17].

It has been suggested that non-eosinophilic asthmatics are poorly responsive to inhaled corticosteroids [8, 18]. One interesting finding of our study is the fact that the so called “non-eosinophilic phenotypes” (neutrophilic and paucigranulocytic phenotypes) actually had more sputum eosinophils than healthy subjects, pointing to a low grade eosinophilic inflammation in these phenotypes. This could explain the fact that some patients with non-eosinophilic asthma responded to inhaled corticosteroids in the study of Cowan et al. [19], even though a limitation of this study was the absence of placebo-controlled design. Also of interest is the fact that eosinophilic asthmatics had more absolute sputum neutrophils compared to healthy subjects. It is likely to reflect that pathways leading to the recruitment of eosinophils and neutrophils in the airways may co-exist or act through a same effector in asthma [20].

It is noteworthy that absolute sputum lymphocyte counts were increased when there was significant eosinophilic infiltration. Although little attention has been paid to lymphocytes in sputum so far because of their small number (representing approximately 1–2 %), our finding is in keeping with the role of lymphocytes in organizing eosinophilic inflammation, a concept which has been established by bronchial biopsy and bronchoalveolar lavage analyses [21, 22].

A particularly low absolute number of macrophages was observed in neutrophilic asthma. Further to this decreased amount of macrophages in the airways, Simpson et al. [23] described an impaired capacity of sputum macrophages to phagocytose apoptotic material in non-eosinophilic asthma, which may explain chronic inflammation and accumulation of airway neutrophils.

In keeping with epithelial alteration in asthma [24], we found an increased epithelial cell number in sputum from eosinophilic and paucigranulocytic asthma. By contrast, epithelial cell count was particularly low in the groups displaying intense neutrophilic inflammation. The presence of epithelial cells in sputum samples may reflect epithelial desquamation. Therefore, it is somewhat surprising to find lower epithelial cell counts in neutrophilic asthma. Our clinical feeling is that the cough effort during sputum induction may favor epithelial shedding and our experience is that effort to produce sputum is less when patients have intense neutrophilic inflammation, which may prevent epithelial cell desquamation.

In contrast to COPD [25], little attention has been paid to systemic inflammation in asthma, so far. Our data show striking elevation of total blood leukocyte counts in all asthmatic phenotypes compared to healthy subjects. There was however no difference between asthma phenotypes in this respect, which is in keeping with the recent study of Zhang et al. [11]. Although raised circulating leukocytes might have been expected in patients with eosinophilic, neutrophilic and mixed granulocytic asthma, this finding was somewhat more surprising in paucigranulocytic asthma.

As previously shown [3, 11], patients with a raised sputum eosinophilic inflammation also had the highest levels of blood eosinophils (absolute values or percentages). Moreover, we found that the highest levels of absolute blood neutrophils were observed in neutrophilic and mixed granulocytic asthma, although the results did not reach statistical significance in this last phenotype, probably due to the small number of patients. Interestingly, paucigranulocytic asthmatics also had raised absolute circulating eosinophils and neutrophils compared to healthy subjects. Overall it points to a granulocytic inflammation in all asthma phenotypes.

Like in sputum, absolute lymphocyte counts were increased in blood of patients with eosinophilic asthma. Interestingly, levels of absolute blood basophils were also higher in these patients, which is consistent with a previous report showing a concomitant increase in circulating eosinophils and basophils after allergen inhalation in atopic asthmatics [26].

Supporting the concept of low grade systemic inflammation in asthma, we found a mild but significant increase in fibrinogen levels in eosinophilic and neutrophilic asthma, while increase in CRP was essentially observed in asthmatics with intense airway neutrophilic infiltration, as previously shown [27].

The group of paucigranulocytic asthmatics may be considered heterogeneous as almost two third of the patients were receiving ICS, which may have attenuated an airway inflammation initially present before the start of the treatment. Therefore, it is interesting to notice that mild airway and systemic eosinophilic inflammation, together with raised circulating lymphocyte number, were still present in those patients not treated with ICS. One limitation of our study is the retrospective design that precludes any further phenotypic characterization of circulating lymphocytes.

Several interventional studies have shown that ICS therapy decreases sputum eosinophils [28, 29] and increases sputum neutrophils [19]. In our study, we did not find any statistically significant difference in these sputum cells between patients treated and not treated with ICS. However, because of its cross-sectional design, our study does not question the impact of ICS on eosinophil and neutrophil counts. We believe that the persistence of significant sputum eosinophilia in ICS treated asthmatics reflects the severity of the inflammatory process in these patients.

Paucigranulocytic asthma is defined as a sputum eosinophil count <3 % in our study. Others have proposed a cutoff at 1.01 % [2]. When choosing this cutoff in our patients, we increase the proportion of eosinophilic asthma from 42 to 51 % and we correspondingly decrease the proportion of paucigranulocytic asthma from 38 to 29 %. With this cutoff at 1.01 %, the group of paucigranulocytic asthmatics still displayed signs of airway and systemic inflammation, although they did not show increased circulating blood eosinophils anymore. It is interesting to postulate that the paucigranulocytic phenotype may be composed of some asthmatics presenting low grade inflammation and others without any sign of airway and systemic inflammation.

Some studies have shown that the eosinophilic inflammation is somewhat related to atopy [3, 30]. Our data suggest that the eosinophilic trait observed in all asthma phenotypes is independent of the atopic status with non-atopic asthmatics not displaying statistically different sputum and blood eosinophil counts from their atopic counterparts.

There was no significant change in the proportion of inflammatory phenotypes according to the smoking status. However, in contrast to sputum eosinophils, sputum neutrophil count appears to be partly associated with the cumulative smoking history expressed as pack-years. Previous studies failed to find this relationship [31, 32] but they were performed on a limited number of patients. This association remains however rather limited based on our results, where pack-years can only account for 5 % of the neutrophil variability.

It is known that asthma inflammatory phenotypes may somewhat vary over time [2, 33]. One limitation of our study is its cross-sectional design with sputum analyzed at only one time point.

## Conclusions

Overall, this study demonstrates that a significant eosinophilic inflammation is present across all categories of asthma, and that paucigranulocytic asthma may be seen as a low grade inflammatory disease. Whether steroid-naïve or a part of steroid-treated paucigranulocytic asthmatics may do without ICS should be investigated in long term trials, but our data incite to be cautious in clinical practice for now.

### Ethics approval and consent to participate

This retrospective study was conducted with the approval from the ethics committee of the University Hospital of Liege (Reference 2015/193). Informed consents were obtained from healthy subjects. As for asthmatic patients, all procedures were performed in the context of clinical practice and the retrospective data collection was conducted with the approval from the above-mentioned ethics committee.

### Consent for publication

Not applicable.

### Availability of data and material

The data supporting our results are presented within the article (and its Additional file 1: Table S1).

## Additional file

Additional file 1: Table S1. Sputum and blood cell counts of eosinophilic phenotypes classified by ICS treatment and healthy subjects. (DOCX 29.3 kb)

## Abbreviations

BMI: body mass index
CRP: C-reactive protein
FENO: fractional exhaled nitric oxide
FEV_1_: forced expiratory volume in 1 second
FVC: forced vital capacity
ICS: inhaled corticosteroid
LABA: long-acting β2-agonist
LTRA: leukotriene receptor antagonist
OCS: oral corticosteroid
PC20M: provocative concentration of methacholine causing a 20 % fall in FEV_1_
