# Supplementary material for: Detailed analysis of sputum and systemic inflammation in asthma phenotypes: are paucigranulocytic asthmatics really non-inflammatory?
Source: BMC Pulm Med. 2016 Apr 5;16:46. doi: 10.1186/s12890-016-0208-2 (PMC4820945; doi:10.1186/s12890-016-0208-2)
Supplement: Additional file 1: Table S1. — Sputum and blood cell counts of eosinophilic phenotypes classified by ICS treatment and healthy subjects. (DOCX 29.3 kb) [file 12890_2016_208_MOESM1_ESM.docx]

**Additional file 1: Sputum and blood cell counts of eosinophilic phenotypes classified by ICS treatment and healthy subjects**

|  | **Eosinophilic asthma,**  **not treated with ICS** | **Eosinophilic asthma,**  **treated with ICS** | **ICS-**  **vs**  **ICS+** | **Mixed granulocytic asthma,**  **not treated with ICS** | **Mixed granulocytic asthma,**  **treated with ICS** | **ICS-**  **vs**  **ICS+** | **Healthy subjects** |
| --- | --- | --- | --- | --- | --- | --- | --- |
| **N** | 123 | 227 | - | 13 | 18 | - | 194 |
| **Sputum total non-squamous cell count (x 106/g)** | 1.12 (0.47-1.92)^‡^ | 1.38 (0.61-3.18)^‡^ | NS | 1.97 (0.73-7.89)^‡^ | 4.71 (1.75-15.10)^‡^ | NS | 0.64 (0.37-1.30) |
| **Sputum viability (%)** | 66 (47-78) | 62 (50-74) | NS | 77 (66-86) | 82 (77-85)^‡^ | NS | 67 (53-78) |
| **Sputum squamous cells (%)** | 16 (7-31) | 13 (5-22)^‡^ | NS | 7 (4-23) | 4 (1-14)^‡^ | NS | 18 (10-31) |
| **Sputum eosinophils (x 103/g)** | 180 (53-436)^‡^ | 217 (68-828)^‡^ | NS | 102 (44-379)^‡^ | 442 (76-808)^‡^ | NS | 0 (0-2) |
| **Sputum neutrophils (x 103/g)** | 283 (132-571) | 374 (135-1003)^‡^ | NS | 1581 (663-7448)^‡^ | 3959 (1330-12714)^‡^ | NS | 210 (63-563) |
| **Sputum macrophages (x 103/g)** | 270 (131-571) | 289 (153-653) | NS | 171 (77-380) | 305 (77-607) | NS | 285 (120-568) |
| **Sputum lymphocytes (x 103/g)** | 20 (5-50) | 18 (5-54) | NS | 15 (0-47) | 23 (1-91) | NS | 10 (4-26) |
| **Sputum epithelial cells (x 103/g)** | 33 (14-81) | 69 (21-162)^‡^ | 0.0034 | 10 (4-23) | 31 (0-121) | NS | 35 (12-86) |
| **Blood leukocytes (x103/µL)**^a^ | 7.16 (5.83-8.28)^‡^ | 7.92 (6.94-9.33)^‡^ | 0.0001 | 7.19 (5.80-7.96) | 8.93 (7.15-11.53)^‡^ | NS | 6.12 (5.03-7.37) |
| **Blood eosinophils (/µL)**^a^ | 270 (192-463)^‡^ | 345 (209-532)^‡^ | NS | 287 (282-397)^‡^ | 342 (211-632)^‡^ | NS | 106 (68-172) |
| **Blood neutrophils (/µL)**^a^ | 3529 (2912-4561) | 4326 (3570-5321)^‡^ | 0.0001 | 3766 (2801-4720) | 4648 (3373-7206) | NS | 3416 (2610-4188) |
| **Blood monocytes (/µL)**^a^ | 489 (387-638)^‡^ | 518 (405-680)^‡^ | NS | 545 (361-763) | 564 (485-779)^‡^ | NS | 399 (330-550) |
| **Blood lymphocytes (/µL)**^a^ | 2347 (1934-2768)^‡^ | 2368 (1911-2868)^‡^ | NS | 2266 (2088-2781) | 2013 (1767-3381) | NS | 2009 (1611-2386) |
| **Blood basophils (/µL)**^a^ | 41 (29-61) | 44 (29-65)^‡^ | NS | 40 (24-49) | 39 (32-69) | NS | 34 (24-46) |

^a^ Data available for 96 healthy subjects

^‡^
lood neutrophils ic inflomme seuil?ernière question, je voudrais faire les comparaisons suivantes:
 3:oupes ("p<0.0042, comparison with healthy subjects

Abbreviation: ICS, inhaled corticosteroid
